# Fluctuation in residual strain and dissipated energy of saturated sandstone under tiered cyclic loading

**DOI:** 10.1371/journal.pone.0236335

**Published:** 2020-07-24

**Authors:** Changbao Jiang, Guojian Cheng, Minke Duan, Yufei Chen, Yang Yang, Qinrong Kang

**Affiliations:** 1 State Key Laboratory of Coal Mine Disaster Dynamics and Control, Chongqing University, Chongqing, China; 2 China Coal Technology Engineering Group Chongqing Research Institute, Chongqing, China; 3 Department of Geology and Petroleum Geology, School of Geosciences, University of Aberdeen, Aberdeen AB24 3UE, United Kingdom; 4 School of Xingfa Mining and Civil Engineering, Wuhan Institute of Technology, Wuhan, Hubei, China; China University of Mining and Technology, CHINA

## Abstract

To study the influence of cyclic stress on the nonlinear behavior of saturated sandstone, the residual strain properties and energy dissipation characteristics of the sandstone under tiered cyclic loading were experimentally investigated. The axial/radial residual deformation and energy dissipation characteristics of sandstone at different cyclic stress stages were analyzed in detail. By combining the mathematical statistics, fluctuation coefficients of the residual strain and energy dissipation, and correlation coefficients of axial/radial residual strain and energy dissipation were defined to describe the process. It was determined that these newly defined physical variables were closely related to the elastic-plastic state (or instability failure state) of the rock.

## Introduction

Granular materials such as soils, rocks, and other granular-filling materials, frequently exhibit nonlinear behavior, including noncontinuity [1], anisotropy [2], non-complete elasticity [3], slow dynamic effects [4, 5], stick-slip [6], and dynamic wave hysteresis [7, 8]. This study focuses on the instability of the deformations and dissipation energies during cyclic loading and unloading processes caused by the noncontinuity and anisotropy of sandstone. These properties are clearly reflected in the nonlinear deformation processes of rocks subjected to cyclic loading [9]. When these processes occur in natural environments, geotechnical engineering construction, and long-term operation, rock and soil masses are often subjected to cyclic or even dynamic loading [10–15]. Cyclic loading causes a complex and sudden nonlinear deformation as well as nonuniform damage to the rock [16, 17]. There are some deviations in the predictions of deformation and failure for nonlinear rock. This is because in the traditional method, rock is regarded as a linear elastic material. Therefore, the study of the nonlinear behavior of rock under cyclic loading is necessary for a better understanding of the damage and failure mechanisms of rock.

In regard to the nonlinear behavior of rock under cyclic loading, many scholars have conducted experiments with different rock types [9–27]. For example, Li et al. [9] established a damage model based on the nonlinear behavior of rocks, and considered the initial damage from fractured rocks from the perspective of energy dissipation. Tong et al. [18] used a Split–Hopkinson pressure bar (SHPB) system to study the nonlinear dynamics of sandstone under different external confining pressures, by exploring the higher harmonics generated by impact loading. David et al. [19] corrected Walsh's model for the effects of cracks on the uniaxial stress-strain curves of rock, and provided a micromechanical explanation for nonlinear and hysteretic stress-strain behavior. Bagde et al. [20, 21] conducted uniaxial cyclic loading tests on sandstone under different amplitudes and frequencies. Effects of the strain, amplitude, and frequency of cyclic loading on the fatigue and nonlinear characteristics of sandstone were studied. Araei et al. [22] experimentally investigated the influence of the initial stress state and loading rate on the nonlinear and hysteretic stress-strain curves of rock under uniaxial cyclic loading. The main focus in the above studies regarding nonlinear characteristics under cyclic loads is hysteresis. However, the volatility of the physical properties of rock under cyclic loading has rarely been studied, especially the fluctuations of residual strain and dissipated energy, and the correlations of axial/radial residual strains and energy dissipations in each cycle under tiered cyclic loading. In addition, the effect of cyclic loading on a rock specimen is based on the cumulative effects during each cycle [23]. The residual deformation and dissipated energy can reflect the fatigue damage process during cyclic loading. Thus, a study of the fluctuations of axial/radial residual deformations and dissipated energy under cyclic loading and their correlations will contribute to understanding the failure mechanism of rock under cyclic fatigue, and to predicting the failure instability of rock.

As rocks are composed of many intimate mineral grains forming a solid frame, the presence of intercrystalline cracks and microstructure boundaries is unavoidable [28]. It is this granular microstructure that causes the fluctuating and nonlinear responses of the rock under external stress [29]. This fluctuating and nonlinear behavior of rock is well known by rock mechanics researchers. In this work, the deformation properties and energy dissipation characteristics of saturated sandstone were experimentally studied under tiered cyclic loading. As rock is a natural heterogeneous material, the residual deformation and dissipated energy show evident fluctuations during the cyclic loading process. The fluctuations of the rock deformation and dissipative energy can reflect the unstable state of the rock during constant-amplitude cyclic loading. Therefore, fluctuation coefficients for residual strain and energy dissipation, correlation coefficients for axial/radial residual strains and energy dissipations were newly defined to describe this process. Finally, the newly defined physical variables were used to identify the elastic plastic state (instability failure state) of the rock. The specific purpose of this study is to evaluate the stability and nonlinear mechanical behavior of an underwater rock mass in the Three Gorges Reservoir area under a cyclic load.

## Experiment setup

### Experimental apparatus

**Fig 1:**
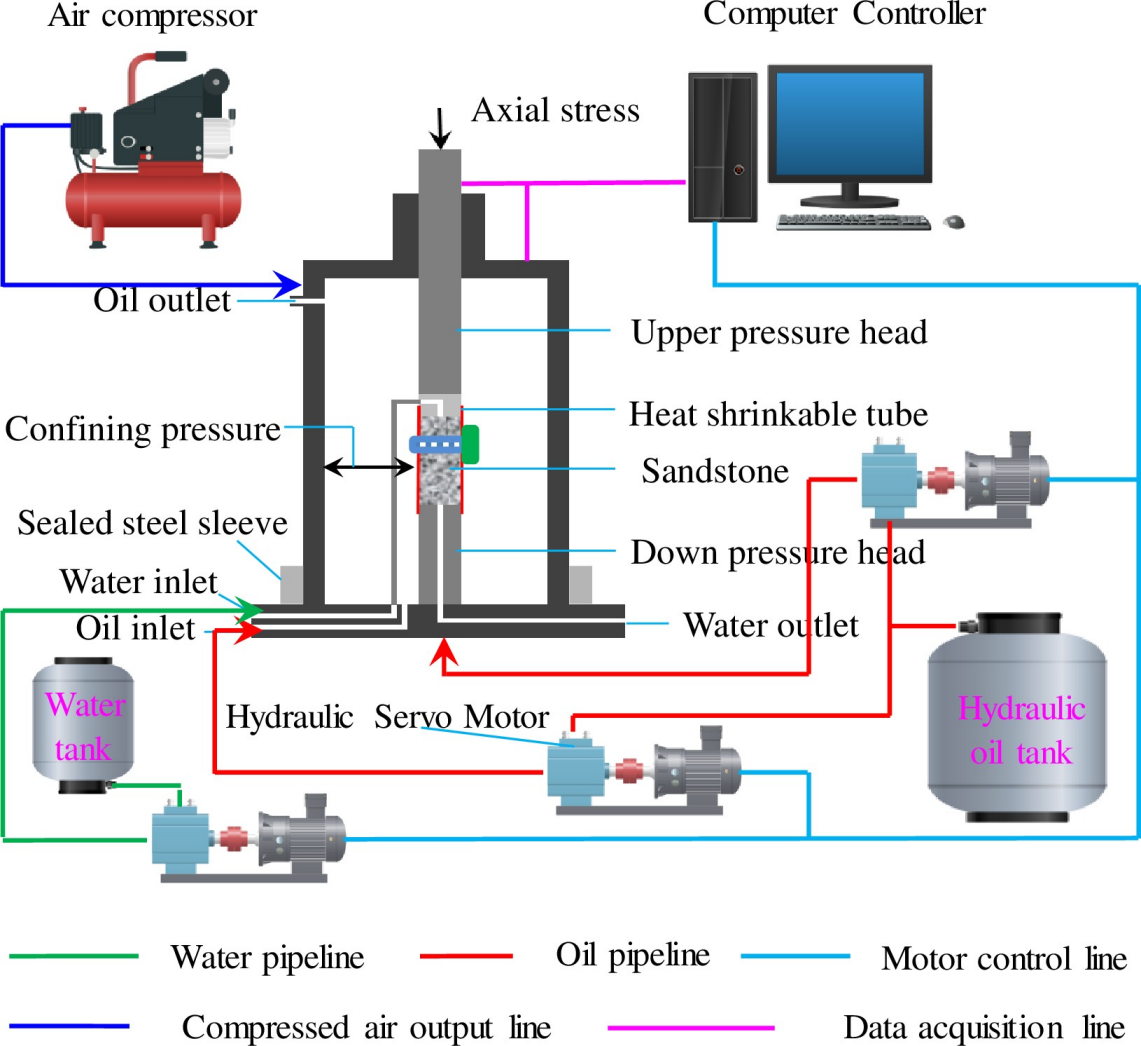
RLW-2000M triaxial creep testing machine.

This experiment was implemented using a RLW-2000M microcomputer controlled coal rock rheometer from Chongqing University, which was produced by Changchun City Chaoyang Test System Co., Ltd. The apparatus was mainly composed of a computer control system, triaxial pressure chamber, axial loading system, confining pressure system, pore water pressure system, and temperature control. The control system adopted an EDC (entire digital control) system imported by the DoLI company of Germany. The system was divided into three independent closed-loop control systems, including systems for axial pressure, confining pressure, and pore pressure. The loading system was a servo motor and ball screw loading system, which can automatically perform the uniaxial compressive strength test, triaxial compressive strength test, cyclic loading test, and rheological test on the rock. It has a maximum axial load of 2000 kN, effective force measurement range of 10–2000 kN, maximum confining pressure of 60 MPa, confining pressure measurement accuracy of ±1%, and maximum pore water pressure of 40 MPa. A diagram of the instrument used for experiment is shown in Fig 1.

### Preparation of sandstone samples

**Fig 2:**
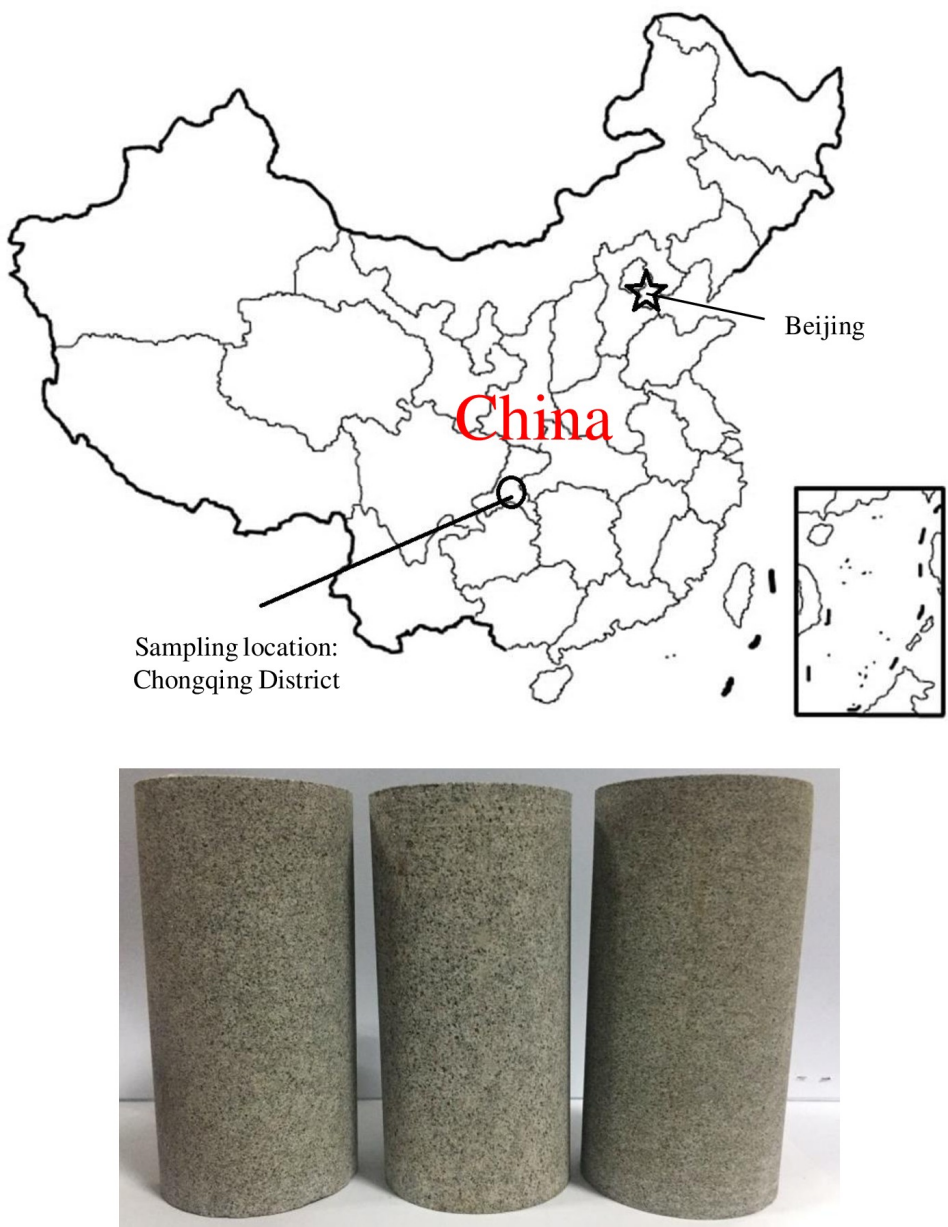
The sandstone samples and sample location.

**Fig 3:**
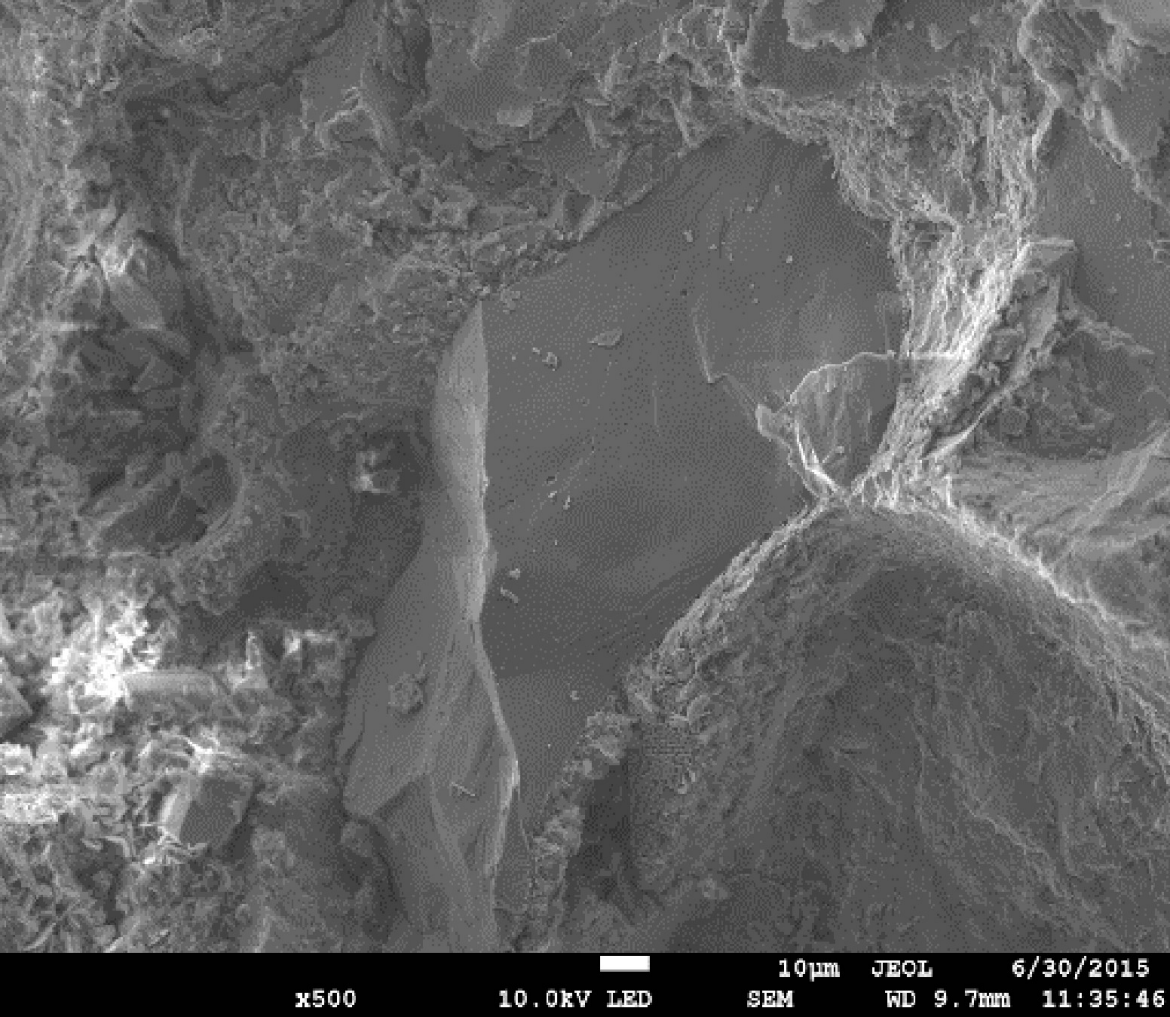
Sandstone internal particle micro-structure scanned by SEM [32].

**Fig 4:**
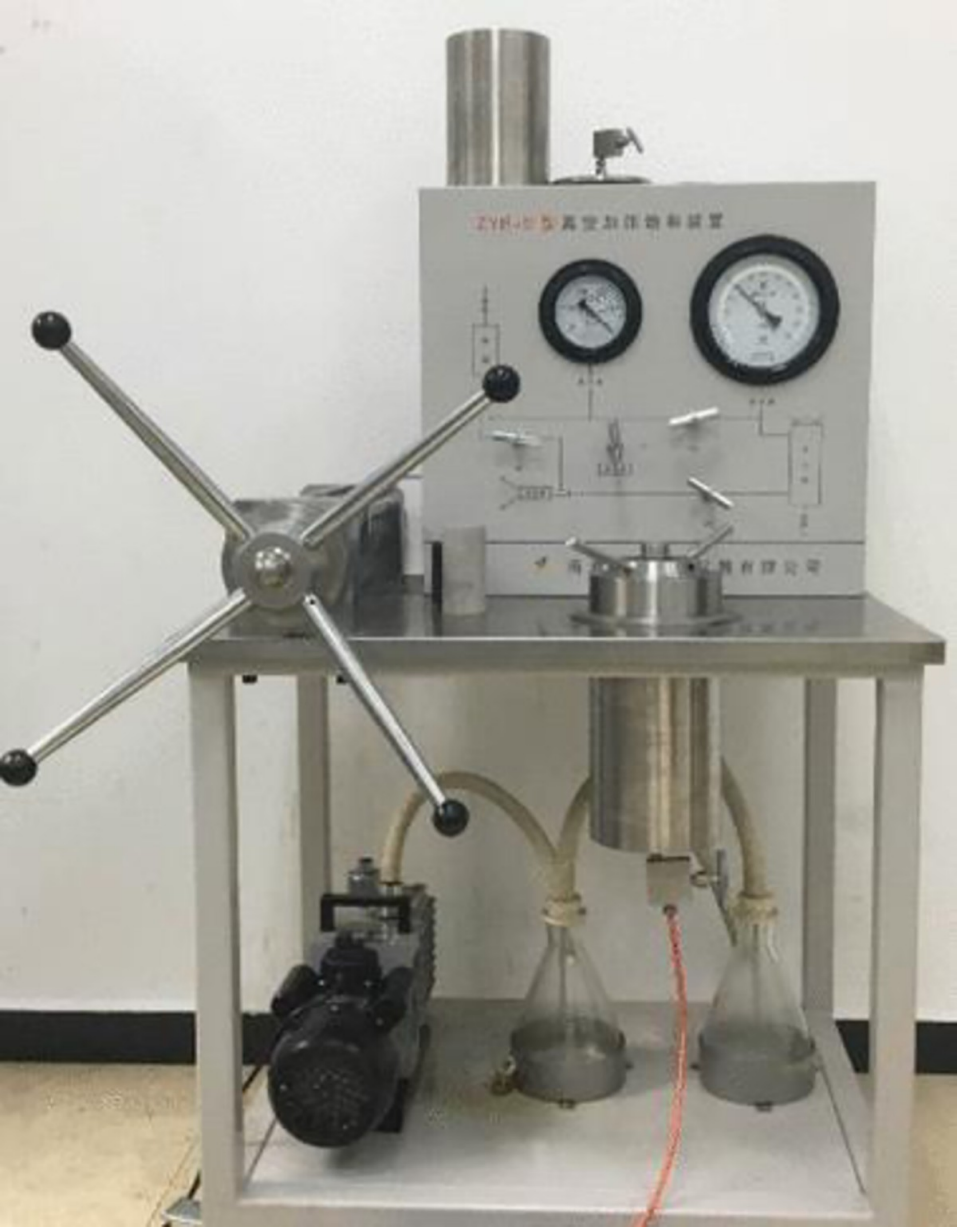
Water-saturated apparatus [33].

The sandstone specimens used in the test were taken from outcrops in the Chongqing District [30], in the Sichuan Basin in the southwest of China (see Fig 2), located in the Three Gorges Reservoir area. The sample collection area in this study is not owned by any company and belongs to public natural resources. The experimental equipment is owned by Chongqing University where the author works; hence, permits were not required. The purpose of this experiment was to evaluate the stability of the underwater rock mass in the Three Gorges Reservoir area under cyclic loading. Therefore, saturated sandstone was selected as the experimental specimen. The study of the mechanical properties of saturated sandstone under repeated engineering disturbances has an important role in ensuring the stability of the rock mass in the Three Gorges Reservoir area. Sandstones are terrigenous fine-grained clastic sedimentary rocks, and are mainly composed of quartz, feldspar and muscovite. The internal structure of sandstone is made up of fine to medium size sand particles with pore structures between the sand particles, cemented mainly by siliceous and carbonate material. According to the ISRM, the samples were processed by wet processing into standard samples with 50 mm in diameter and 100 mm in height. In addition, the sample cross section was cut and polished such that the end face evenness was controlled to within 0.02 mm [31]. The basic physical parameters of the sandstone are listed in Table 1. Fig 3 shows the sandstone internal particle micro-structure, obtained via scanning electron microscopy [32]. As the effects of the pore water pressure were considered in the experiment, it was necessary to prepare a saturated sandstone specimen. A water-saturated apparatus was used to prepare the saturated specimen, as shown in Fig 4. Jiang et al. [33] can be referred to for specific water saturation steps.

**Table 1:** Basic physical parameters of sandstone.

Natural moisture content (%)	Density (g/cm^3^)	Modulus of elasticity (GPa)	Poisson ratio	Cohesive force (MPa)	Internal friction angle (°)
3.8	2.45	13.8	0.27	14.5	42

### Experiment scheme

**Fig 5:**
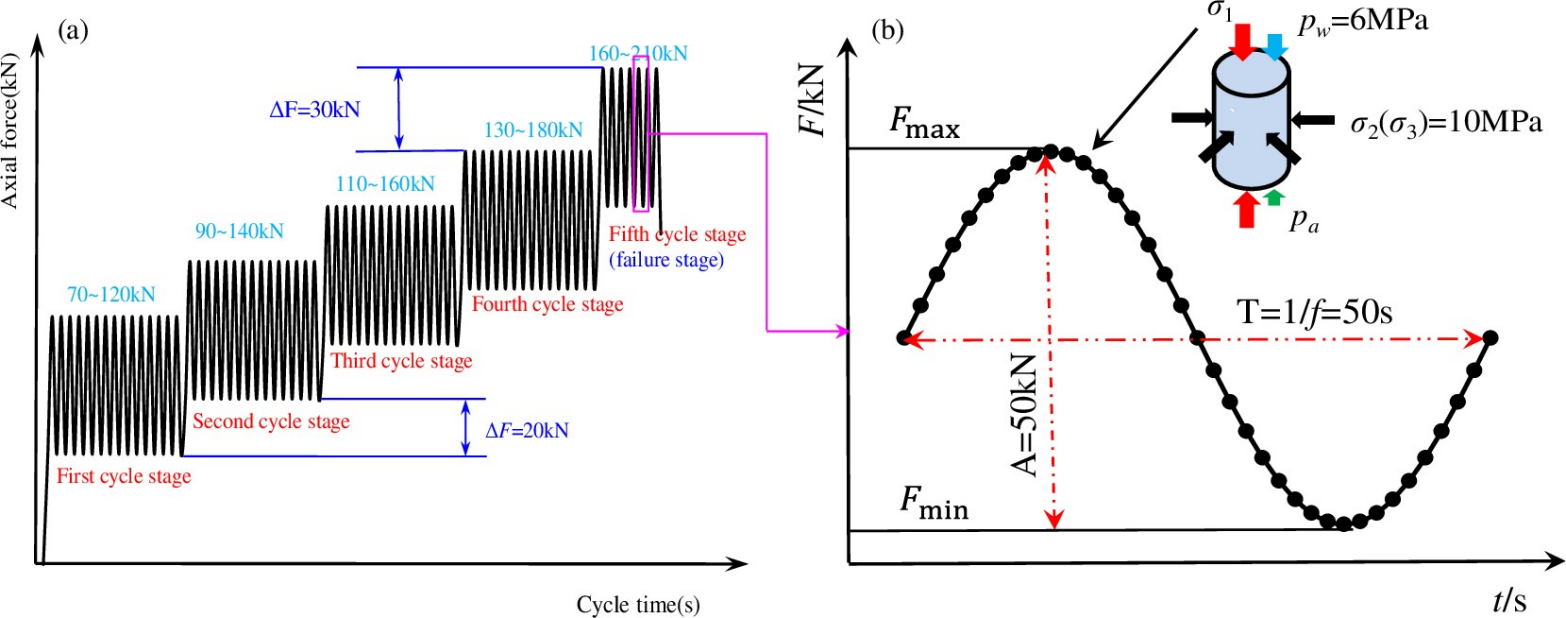
Schematic diagram of tiered cyclic loading and Sketch of sine wave loading (water pressure 6MPa).

In this experiment, saturated sandstone was initially placed into the triaxial pressure chamber. Thereafter, the triaxial pressure chamber was filled with hydraulic oil to apply the confining pressure. After making contact with the axial indenter, an axial force was applied to 20 kN at a speed of 0.1 kN/s. The confining pressure was applied to a hydrostatic pressure of 10 MPa at a speed of 0.05 MPa/s, and the water pressure was loaded to a target value (3 and 6 MPa) at a speed of 0.05 MPa/s. The confining and water pressures were kept constant, and the axial stress continued to be loaded to 70 kN. After the deformation displacement and stability state, the axial stress entered a stage of tiered cyclic loading; the path for the axial stress tiered cyclic loading is illustrated in Fig 5(A). Each cycle stage was numbered, and the final cycle stage was called the failure stage. The cyclic frequency was set to 0.02 Hz, and the cycle number was set to 140 times per cycle stage. The stress amplitude was set to 50 kN for each cycle stage. The axial stress was set to an increment of 20 kN per stage, and continuous cyclic loading was conducted until failure of the sandstone specimen. To smooth the peak and valley of the stress-strain curve from the cyclic loading, a sinusoidal wave was used as the loading waveform in this experiment, as shown in Fig 5(B).

## Experimental results and analysis

In this experiment, the tiered cyclic loading tests were carried out on two saturated sandstone specimens (SS-1 and SS-2) under different water pressures (3 MPa and 6 MPa, respectively). The characteristics of the axial/radial deformations and energy dissipations were compared and analyzed under different cycle stages and different water pressures. By analyzing these value and their correlations, the rock mass deformation instability and damage could be predicted. Table 2 provides the basic experimental data from SS-1 and SS-2. (Note: SS is the abbreviation for saturated sandstone)

**Table 2:** Basic experimental data from sandstone sample.

Sample	Cycle stage	*ξ*_1_(10^−7^)	*ξ*_3_(10^−7^)	*χ*_1_	*χ*_3_	*ρ*_p_	*ρ*_d_
SS-1 (water pressure 3 MPa)	1th	0.589	0.1721	0.0143	0.0395	-0.808	-0.750
	2th	0.239	0.1455	0.0075	0.0152	-0.925	-0.948
	3th	0.485	0.6401	0.0254	0.0395	-0.931	-0.958
	4th	0.766	0.9673	0.0389	0.0452	-0.445	-0.574
	5th	18.557	16.270	0.1291	0.1554	-0.433	-0.521
SS-2 (water pressure 6 MPa)	1th	0.569	0.1074	0.120	0.300	-0.873	-0.896
	2th	0.493	0.0882	0.024	0.035	-0.923	-0.902
	3th	2.734	0.1658	0.145	0.065	-0.956	-0.965
	4th	3.682	0.2256	0.523	0.153	-0.930	-0.925

*ξ*_1_ and *ξ*_3_ are the fluctuation coefficients of Δ*ε*_*p*1_ and Δ*ε*_*p*3_, respectively; *χ*_1_ and *χ*_3_ are the fluctuation coefficients of Δ*U*_*d*1_ and Δ*U*_*d*3_, respectively; *ρ*_*p*_ is the correlation coefficient of Δ*ε*_*p*1_^*ij*^ and Δ*ε*_*p*3_^*ij*^; *ρ*_*d*_ is the correlation coefficient of Δ*U*_*d*1_^*ij*^ and Δ*U*_*d*3_^*ij*^.

### Characteristics of residual strain in sandstone under tiered cyclic loading

**Fig 6:**
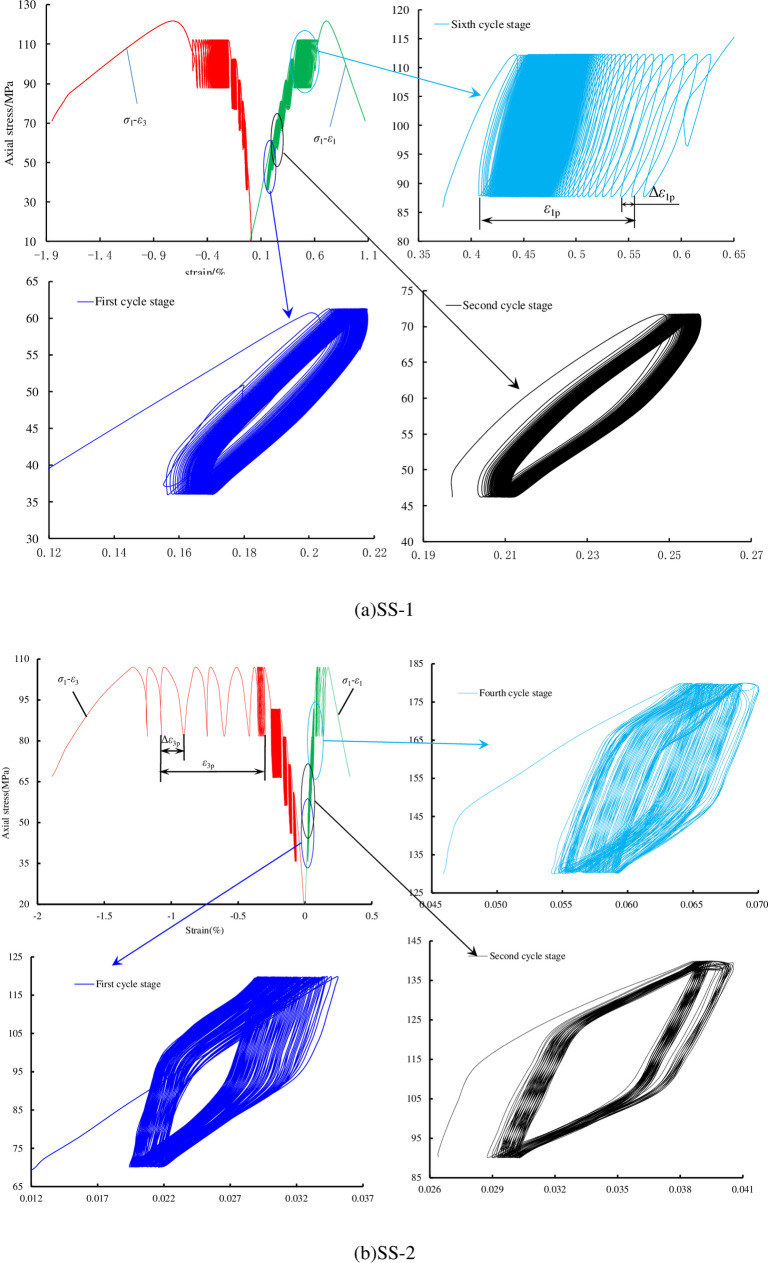
Full stress-strain curves and local enlarged diagrams of sandstone under multilevel cyclic loading.

The full stress-strain curves of the two saturated sandstone specimens under tiered cyclic loading are illustrated in Fig 6. The stress-strain curves of the sandstone specimens under different water pressures were similar during cyclic loading. A large amount of plastic deformation was produced in each sandstone specimen at each cycle stage. With an increase of the cyclic stage, the cyclic stress-strain curve showed characteristics of three different stages, i.e., sparse, then dense, and later sparse again. Owing to the existence of micro-pores, cracks, and soft interfaces in the sandstone, the deformation of the sandstone at the initial cyclic stage was large. With the increase of stress, the sandstone was gradually compacted and the plastic deformation decreased. When the cyclic stress exceeded the fatigue threshold stress of the sandstone, plastic deformation increased.

To analyze the residual strain of the sandstone during the cyclic loading process more precisely, the formulas for calculating the accumulated residual strain and relative residual strain at different cycle stages were defined as follows

$$\left\{ \begin{array}{l} \varepsilon_{p}^{ij}=\varepsilon^{ij}-\varepsilon^{i0}\\ \Delta \varepsilon_{p}^{ij}=\varepsilon^{ij}-\varepsilon^{i(j-1)} \end{array}\right.$$ (1)

where *ε*_*p*_^*ij*^ is the accumulated residual strain at *j*th cycle in the *i*th cycle stage; it includes the accumulated axial residual strain (*ε*_*p*1_^*ij*^), and the accumulated radial residual strain (*ε*_*p*3_^*ij*^). Δ*ε*_p_^*ij*^ is the relative residual strain at the *j*th cycle in the *i*th cycle stage, and also includes the relative axial residual strain (Δ*ε*_*p*1_^*ij*^) and relative radial residual strain (Δ*ε*_*p*3_^*ij*^).

### The change in *ε*_*p*_^*ij*^ under different cyclic stress level

**Fig 7:**
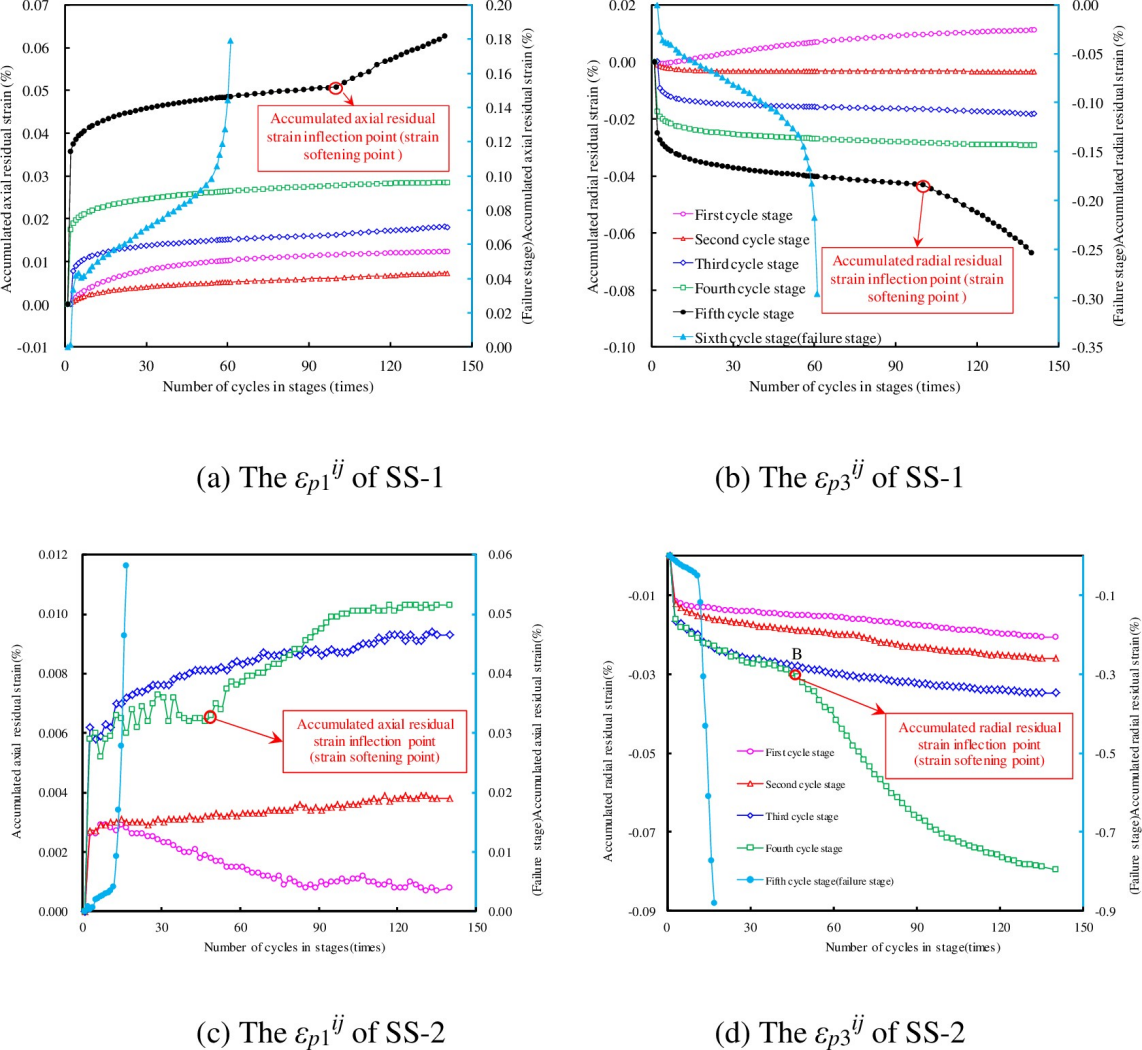
Accumulated residual strain curves at different cycle stages.

The values of *ε*_*p*1_^*ij*^ and *ε*_*p*3_^*ij*^ can be calculated using Eq (1), and the results for SS-1 and SS-2 are shown in Fig 7. It can be seen that the changes in the accumulated residual strain under different water pressures are similar. However, the smaller the water pressure, the smoother the residual strain curve and the smaller the deformation fluctuation. The accumulated residual strain increased with the increase of the cyclic stress (or stage), but it showed different changes in each cycle stage with the increasing cycle number.

1. The change in *ε*_*p*1_^*ij*^: When the cyclic stress is in the elastic stage (i.e., in the first few cycle stages), *ε*_*p*1_^*ij*^ initially increases rapidly, then gradually, and finally becomes stable; when the cyclic stress is in the yield stage (i.e., in the several cycle stages before destruction), *ε*_*p*1_^*ij*^ shows a large increase and fluctuates greatly, and there is an evident inflection point (or strain-softening point), as depicted in Fig 7. In the cyclic destruction stage, the change trend of *ε*_*p*1_^*ij*^ shows an evident acceleration deceleration acceleration process. It increases sharply, and the portion near failure increases linearly.
2. The change in *ε*_*p*3_^*ij*^: The absolute value of *ε*_*p*3_^*ij*^ has the same change as that of *ε*_*p*1_^*ij*^. The evident inflection point also appears in the same cycle stage. As the stresses increase gradually, when the stress reaches the fatigue threshold stress for sandstone, the strain of the sandstone under cyclic loading increases sharply.

### The change in Δ*ε*_*p*_^*ij*^ under different cyclic stress level

**Fig 8:**
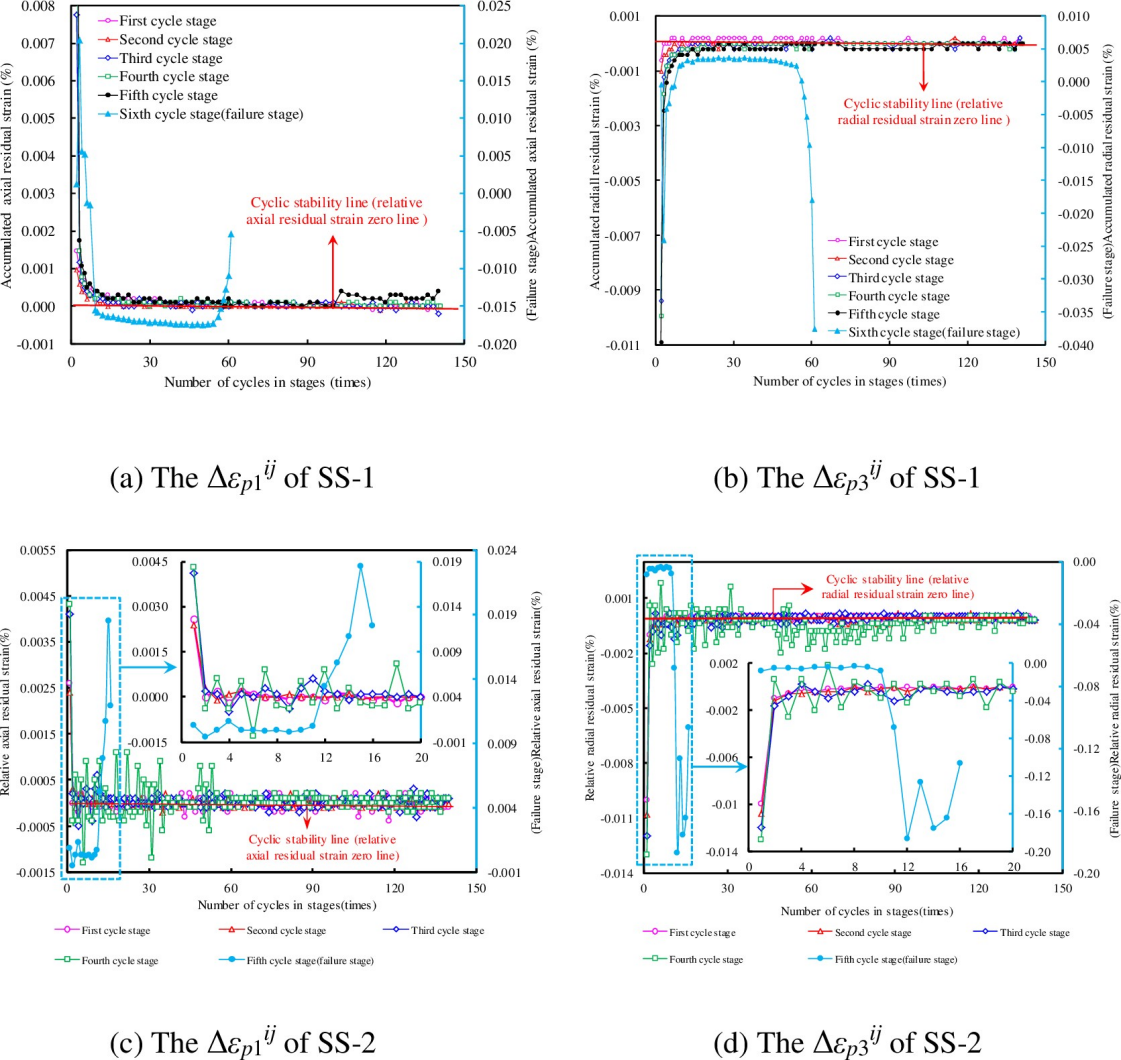
Relative residual strain curve at different cycle stages.

The values of Δ*ε*_*p*1_^*ij*^ and Δ*ε*_*p*3_^*ij*^ at each cycle stage can be calculated using Eq (1), as shown in Fig 8. To clearly observe the relative residual strain of SS-2 in each cycle stage, the Δ*ε*_*p*_^*ij*^ curve for the first 20 cycles in each cycle stage is enlarged. The change in Δ*ε*_*p*_^*ij*^ in the sandstone can be divided into two stages.

1. Before failure stage: In the first 4^*th*^ (or 5^*th*^) cycle stages, and with the increase of cycle number, the values of Δ*ε*_*p*1_^*ij*^ and the absolute value of Δ*ε*_*p*3_^*ij*^ are larger in the initial cycles, and then gradually approach the cyclic stability line (i.e., the 0 line). The relative residual strain fluctuates in the vicinity of the 0 line, even infinitely close to 0. This indicates that the initial residual deformation at each stage is large, then decreases gradually, before finally reaching nearly 0.
2. Failure stage: In the 5^*th*^ (or 6^*th*^) cycle stage, with the increase of cycle number, the values of Δ*ε*_*p*1_^*ij*^ and absolute value of Δ*ε*_*p*3_^*ij*^ begin to increase slowly, and then increases sharply when the samples are near failure.

### Characteristic dissipation energy in sandstone under tiered cyclic loading

In addition to the residual strains, plastic hysteresis loops were also produced in the cyclic stress-strain curves of the sandstone. The residual strain can only reflect the plastic deformation at the unloading point; in contrast, the area of the plastic hysteresis loop is a process quantity which can reflect the deformation of the sandstone during the entire loading-unloading process. The physical implication of the hysteresis loop area is the energy dissipation of the rock during the process of a single loading and unloading [12, 34]. Energy dissipation is an essential property of rock failure and a common measure for analyzing the failure process [24, 35–37] Therefore, it is necessary to conduct a detailed analysis of the energy dissipation under tiered cyclic loading.

During the loading process, rock deformations occur owing to the effects of external forces, and the total energy (*U*) input from an external force to a rock sample is mainly stored as an elastic potential energy (*U*_*e*_) accumulated in the rock mass. A small part of the energy (*U*_*d*_) is lost in various forms of damage dissipation. The elastic potential energy is released when unloading. In this study, this energy was approximately equal to the negative work *U*_r_ done by the external forces during unloading [12, 34] as follows:

$$U=U_{e}+U_{d}\approx U_{r}+U_{d}=\int \sigma_{1}^{+}d\varepsilon_{1}^{+}+2\int \sigma_{3}^{+}d\varepsilon_{3}^{+}$$ (2)

$$U_{r}=\int \sigma_{1}^{-}d\varepsilon_{1}^{-}+2\int \sigma_{3}^{-}d\varepsilon_{3}^{-}$$ (3)

The area of a plastic hysteresis loop represents the single cycle dissipation energy, including the axial dissipation energy and radial dissipation energy [35]. In combination with the definition of a differential, the axial dissipation energy and radial dissipation energy can be calculated as follows [12, 34–37]:

$$U_{d}=U-U_{r}=(\int \sigma_{1}^{+}d\varepsilon_{1}^{+}-\int \sigma_{1}^{-}d\varepsilon_{1}^{-})+2(\int \sigma_{3}^{+}d\varepsilon_{3}^{+}-\int \sigma_{3}^{-}d\varepsilon_{3}^{-})=U_{d1}+U_{d3}$$ (4)

$$\int \sigma d\varepsilon =\sum_{i=1}^{n}\frac{1}{2}(\sigma^{i}+\sigma^{i-1})(\varepsilon^{i}-\varepsilon^{i-1})$$ (5)

where *U*_*d*1_ and *U*_*d*3_ are the axial and radial dissipation energy, respectively. *σ*^+^_1_, *ε*^+^_1_, *σ*^-^_1_, and *ε*^-^_1_ are the stress and strain values at each point of the axial stress-strain curve in the loading and unloading stages, respectively, and *σ*_3_ is radial stress. *ε*^*+*^_3_ and *ε*^*-*^_3_ are the radial strain at loading and unloading stages, respectively. Δ*U*_*d*_ indicates the single cycle dissipated energy.

**Fig 9:**
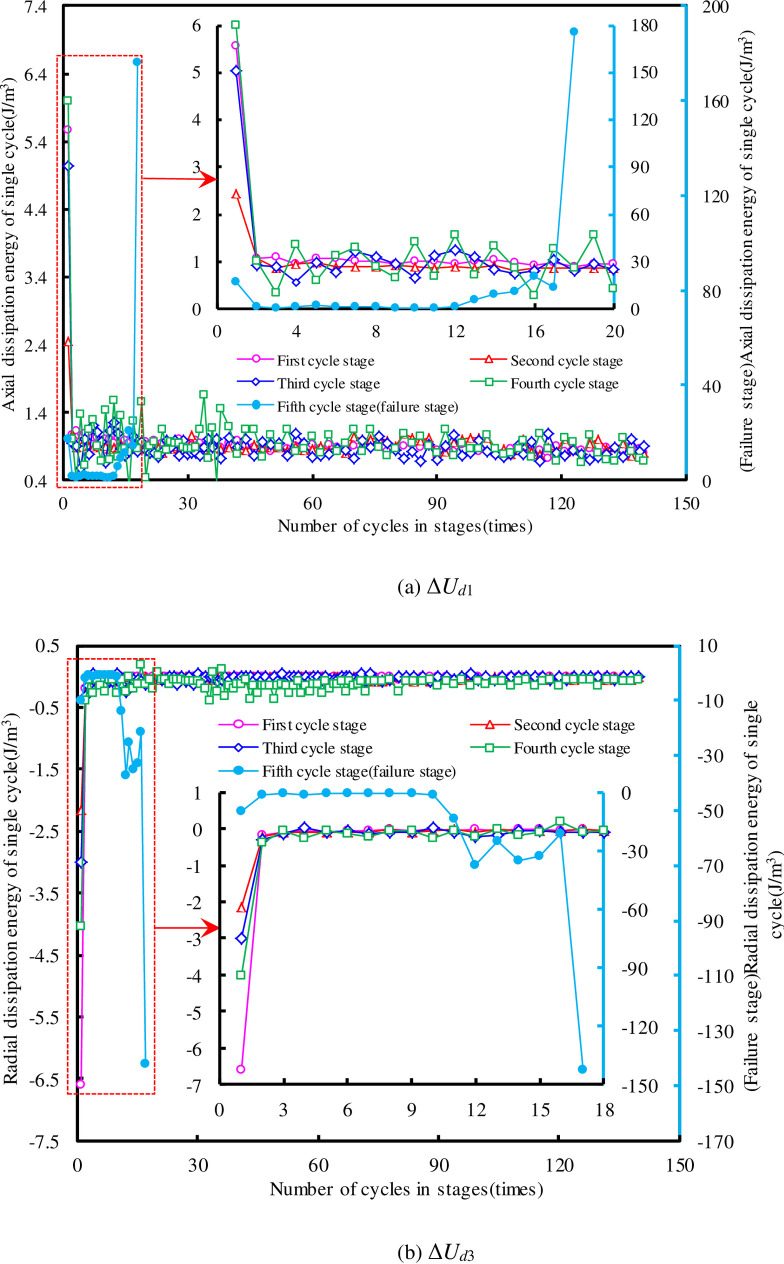
The curve of Δ*U*_*d*_ in the SS-2 at different cycle stages.

As the dissipative energy laws of the two sandstone specimens are similar (the smaller the water pressure, the smoother the dissipation energy curve.), only the dissipative energy evolution law of SS-2 was analyzed in detail. The changes of Δ*U*_*d*1_ and Δ*U*_*d*3_ in SS-2 are shown in Fig 9. It can be seen that Δ*U*_*d*3_ is negative, indicating that the specimen do work to hydraulic oil in the radial direction during cycle loading. The curve of Δ*U*_*d*_ for the first 20 cycles at each cycle stage is also enlarged. There were similarities between the variation trends of Δ*U*_*d*_ and Δ*ε*_*p*_. The change in Δ*U*_*d*_ can also be divided into two stages (i.e., a pre-destruction stage and destruction stage).

### Fluctuations of Δ*ε*_*p*_ and Δ*U*_*d*_ under tiered cyclic loading

**Fig 10:**
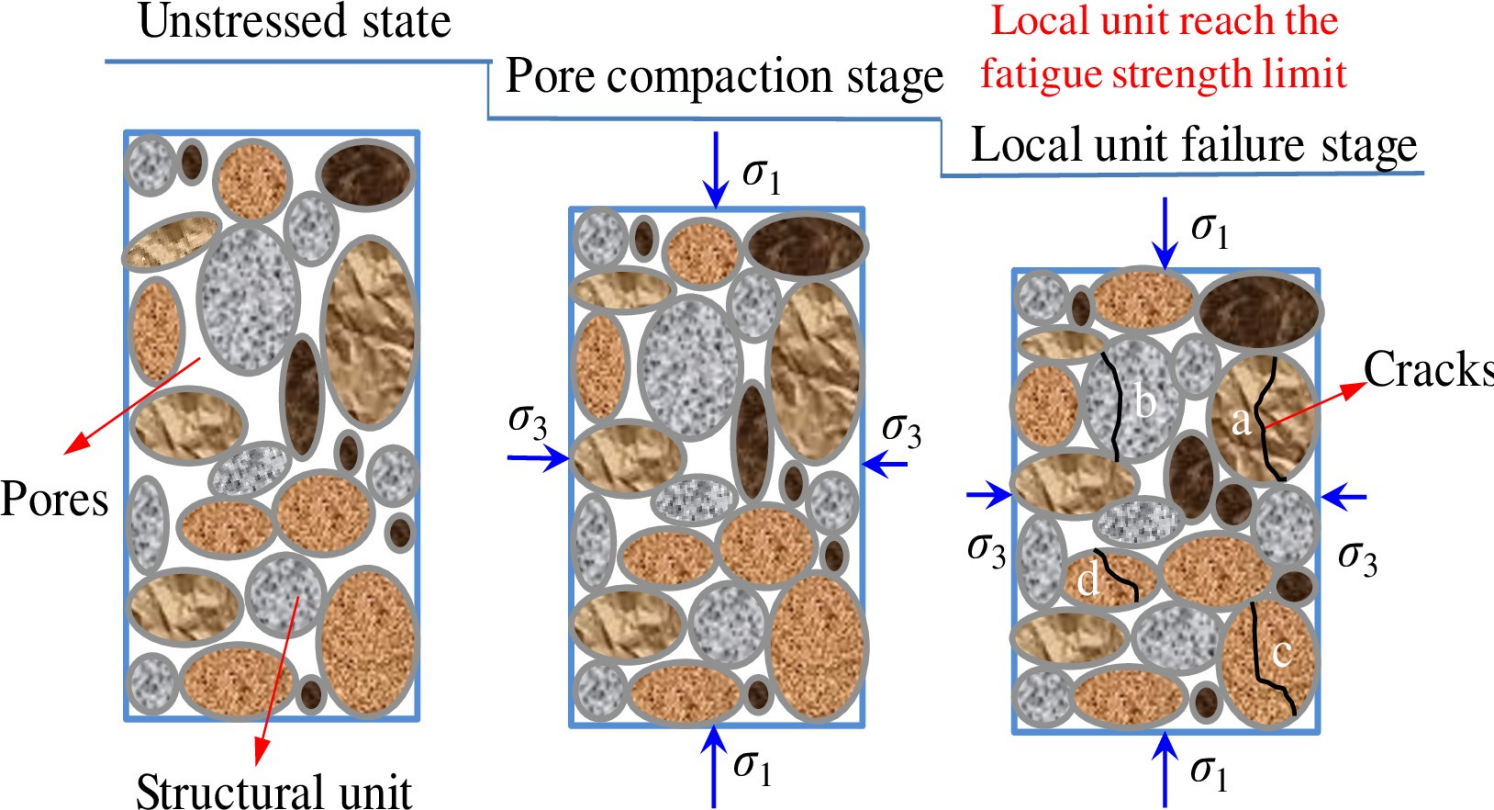
Schematic diagram of deformation and failure of sandstone structural units under external force.

From Figs 8 and 9, it is observed that the curves of Δ*U*_*d*_ and Δ*ε*_*p*_ show some fluctuation in each cycle stage before the destruction stage; this phenomenon has also been found in other studies [38–42]. There are many explanations for this phenomenon, but it is generally accepted that a rock mass is composed of micro-structural elements (see Fig 10). Each micro-structural element has fatigue strength limit. When the cyclic stress reaches the failure strength limit of each micro-structural element, local failure occurs and the residual deformation of rock suddenly increases [9, 23]. However, other researchers believe that sandstone subjected to cyclic stress displays complex stress-strain characteristics of nonlinearity and hysteresis, owing mainly to nonlinear deformations and the frictional sliding of grain contacts [40–42]. The phenomenon of unstable deformation (or unstable damage) of sandstone exactly reflects that sandstone is a natural product of heterogeneity and anisotropy [43, 44]. The stability of the rock can be predicted by analyzing the fluctuations of the deformation and dissipation energy.

By applying the theory of variance in mathematical statistics, the fluctuation coefficient of Δ*ε*_*p*_ was defined as the variance of Δ*ε*_*p*_ in each cycle stage before failure. The fluctuation coefficient of Δ*ε*_*p*_ was calculated as follows:

$$E(\Delta \varepsilon_{p}^{ij})=\sum_{j=1}^{140}(\Delta \varepsilon_{p}^{ij})/140$$ (6)

$$\xi =D(\Delta \varepsilon_{p}^{ij})=E\left\{{\left[\Delta \varepsilon_{p}^{ij}-E(\Delta \varepsilon_{p}^{ij})\right]}^{2}\right\}$$ (7)

where *E*(Δ*ε*_p_^*ij*^) is the expected value of Δ*ε*_*p*_ in the *i*th cycle stage before failure. *ξ* is the variance of Δ*ε*_*p*_ in the *i*th cycle stage before failure. *ξ*_1_ and *ξ*_3_ are the fluctuation coefficients of Δ*ε*_*p*1_ and Δ*ε*_*p*3_, respectively.

**Fig 11:**
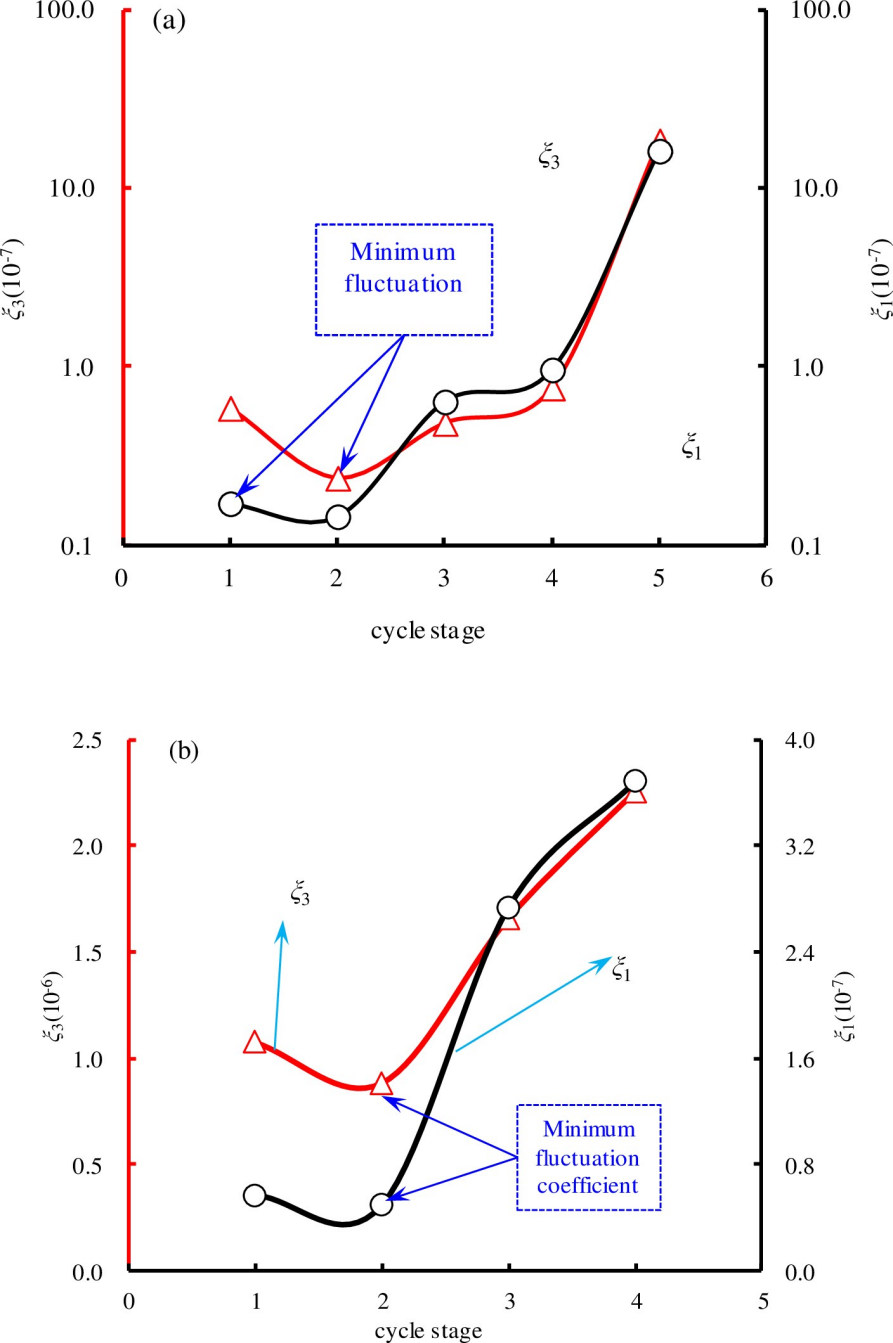
Fluctuation coefficient curve of relative residual strain: (a) SS-1; (b) SS-2.

The values of *ξ*_1_ and *ξ*_3_ are illustrated in Fig 11; they exhibit the same trend with the increase of cycle stage, i.e., both initially decrease and then increase. In the initial cycle stage, owing to the large number of micro-pores, cracks, and weak zones in the original rock mass, unstable deformation easily occurs under stress. With an increase of cyclic stress, the pore fracture is gradually compacted, and the rock mass tends to be elastic. Thus, the fluctuation of Δ*ε*_*p*_ decreased in second cycle stage, and the variance of Δ*ε*_*p*_ was close to 0. As the cyclic stress continued to increase, new pores and fissures gradually expanded, and local failures resulted in unstable deformations. The fluctuation of Δ*ε*_*p*_ increased rapidly, and the deformation of the rock mass became increasingly unstable. This show that the deformation has great fluctuations when the rock mass tends to instability. The characteristics of Δ*ε*_*p*_ can be used to predict whether rock mass failure will occur during cyclic loading.

In the same way, the fluctuation coefficient of Δ*U*_*d*_ was calculated as follows:

$$E(\Delta U_{d}^{ij})=\sum_{j=1}^{140}(\Delta U_{d}^{ij})/140$$ (8)

$$\chi =D(\Delta U_{d}^{ij})=E\left\{{\left[\Delta U_{d}^{ij}-E(\Delta U_{d}^{ij})\right]}^{2}\right\}$$ (9)

where *E*(Δ*U*_*d*_^*ij*^) is the expected value of the single cycle energy dissipation in the *i*th cycle stage before failure. *χ* is variance value of the single cycle energy dissipation in the *i*th cycle stage before failure. *χ*_1_ and *χ*_3_ are the fluctuation coefficients of Δ*U*_*d*1_ and Δ*U*_*d*3_, respectively.

**Fig 12:**
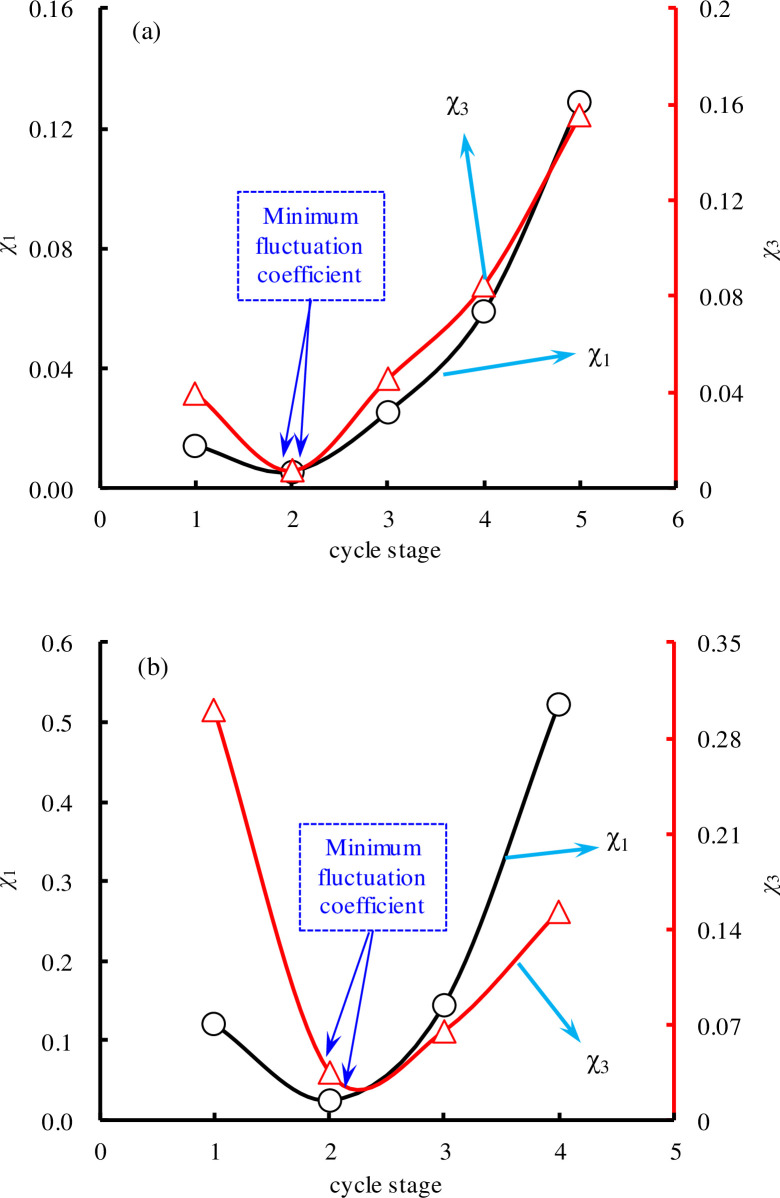
Fluctuation coefficient of dissipation energy in single cycle: (a) SS-1; (b) SS-2.

As shown in Fig 12, the change trend of *χ* is the same as the change trend of *ξ*, but *χ* is disproportionate to *ξ*. This shows that there is a certain relationship (but not a linear relationship) between the residual strain and energy dissipation under cyclic loading. *χ*_1_ and *χ*_3_ show the same trend with increasing cycle stage, i.e., initial decrease and then increase. There was some instability in the energy dissipation at the initial stage and near the destruction stage. The characteristics of Δ*U*_*d*_ can also be used as a precursor for predicting rock damage.

### Correlation coefficients of axial/radial residual strains and energy dissipations

From Figs 11 and 12, it can be seen that there is some correlation between the axial and radial residual strains (or energy dissipations); this indicates that the axial and radial physical variables are interrelated, and interact with each other. The relationship between axial and radial strains is most commonly expressed by Poisson's ratio. For perfectly elastic materials, the Poisson's ratio is a constant, and shows that the relationship between the axial and radial strains is linear. For sandstone, the Poisson's ratio varied under stress loading, indicating that the relationship between the axial and radial strains is nonlinear. To observe correlation strength between the axial and radial relative residual strains, the correlation coefficients of Δ*ε*_*p*1_ and Δ*ε*_*p*3_ are defined as follows:

$$Cov(\Delta \varepsilon_{p1}^{ij},\Delta \varepsilon_{p3}^{ij})=E\left\{\left[\Delta \varepsilon_{p1}^{ij}-E(\Delta \varepsilon_{p1}^{ij})\right]\left[\Delta \varepsilon_{p3}^{ij}-E(\Delta \varepsilon_{p3}^{ij})\right]\right\}$$ (10)

$$\rho_{p}=Cov(\Delta \varepsilon_{p1}^{ij},\Delta \varepsilon_{p3}^{ij})/\sqrt{\xi_{1}\xi_{3}}$$ (11)

where *Cov*(Δ*ε*_*p*1_^*ij*^, Δ*ε*_*p*3_^*ij*^) is the covariance of Δ*ε*_*p*1_^*ij*^ and Δ*ε*_*p*3_^*ij*^; *ρ*_*p*_ is the correlation coefficient of Δ*ε*_*p*1_^*ij*^ and Δ*ε*_*p*3_^*ij*^, and can reflect the correlation strength between the axial residual strain and radial residual strain in each cycle stage.

In the same way, the correlation coefficients of the single cycle axial and radial energy dissipations are defined as follows:

$$Cov(\Delta U_{d1}^{ij},\Delta U_{d3}^{ij})=E\left\{\left[\Delta U_{d1}^{ij}-E(\Delta U_{d1}^{ij})\right]\left[\Delta U_{d3}^{ij}-E(\Delta U_{d3}^{ij})\right]\right\}$$ (12)

$$\rho_{d}=Cov(\Delta U_{d1}^{ij},\Delta U_{d3}^{ij})/\sqrt{\chi_{1}\chi_{3}}$$ (13)

where *Cov*(Δ*U*_*d*1_^*ij*^, Δ*U*_*d*3_^*ij*^) is the covariance of Δ*U*_*d*1_^*ij*^ and Δ*U*_*d*3_^*ij*^; *ρ*_*d*_ is the correlation coefficient of Δ*U*_*d*1_^*ij*^ and Δ*U*_*d*3_^*ij*^, and can reflect the correlation strength between the axial and radial energy dissipation in a single cycle at each cycle stage.

The correlation coefficient *ρ* has the following properties: |*ρ*|≤1. if and only if there is a linear relationship between Δ*ε*_*p*1_^*ij*^ and Δ*ε*_*p*3_^*ij*^ (or Δ*U*_*d*1_^*ij*^ and Δ*U*_*d*3_^*ij*^), that is Δ*ε*_*p*3_^*ij*^ = *a*+*b*Δ*ε*_*p*1_^*ij*^ (or Δ*U*_*d*1_^*ij*^ = *a*+*b*Δ*U*_*d*3_^*ij*^), |*ρ*| = 1, and the following holds:

$$\rho =\left\{ \begin{array}{l} 1,b>0,\\ -1,b<0. \end{array}\right.$$ (14)

Through the above mentioned properties, we can see that when *ρ* = ±1, the strongest correlation is observed between the two variables, i.e., a linear correlation. When *ρ* = 0, the two independent variables are not related. *ρ*>0 represents a positive correlation, whereas *ρ*<0 represents a negative correlation.

**Fig 13:**
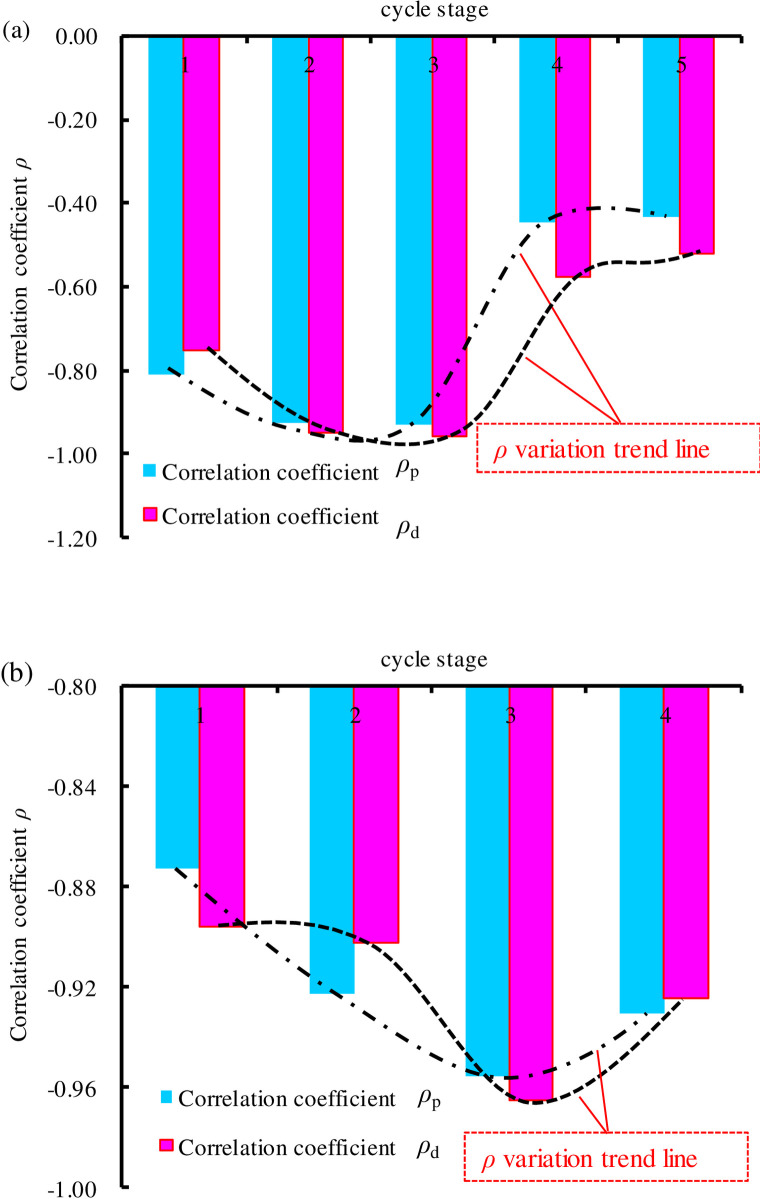
Correlation coefficient curve: (a) SS-1; (b) SS-2.

The values of *ρ*_*p*_ and *ρ*_*d*_ in each cycle stage can be calculated by Eqs (11) and (13). As shown in Fig 13, some results can be obtained, as follow. First, the changes of *ρ*_d_ and *ρ*_*p*_ are similar, so *ρ*_*p*_ can be employed as an example for detailed analysis. When *ρ*_*p*_<0, it indicates that Δ*ε*_*p*1_^*ij*^ and Δ*ε*_*p*3_^*ij*^ have negative correlation. With the increase of cyclic stress, *ρ*_*p*_ decreases first and then increases; in contrast, the correlation intensity increases first, and then decreases. In the 3^*th*^ cycle stage, *ρ*_*p*_ reached a minimum value of -0.956 for SS-1 (or -0.931 for SS-2), i.e., the strongest correlation still did not reach -1, indicating that Δ*ε*_*p*1_^*ij*^ and Δ*ε*_*p*3_^*ij*^ were not linearly related. The mechanical behaviors of sandstone show evident nonlinear characteristics, and the integrity and coordination of the axial and radial deformations change with the stress. This is because sandstone is a natural, heterogeneous, and defective material. It contains a complex pore structure and is not an ideal elastic material. The ratio of the axial strain to the radial strain (i.e. the Poisson ratio) is not a constant but a variable under the action of force. When the stress changes, the correlation between the axial strain and radial strain also changes, and the correlation between the axial strain and the radial strain can exactly reflect the elastic plastic state or integrity of the rock. As illustrated in Fig 13, when the cyclic stress increases, the sandstone material is under constant compaction. The elasticity of the sandstone increased, the plasticity decreased, and the correlation between the axial and radial deformations was enhanced. As the cyclic stress continued to increase and exceeded the fatigue threshold stress, the sandstone fracture expanded. The plasticity of the sandstone increased, the elasticity decreased, and the correlation between the axial and radial deformation decreased. Therefore, the correlation between the axial and radial physical parameters in the rock can also be used as an index for predicting rock instability. In summary, the newly defined physical variables (*ξ*, *χ*, *ρ*) in this study are closely related to the elastic plastic state (or instability failure state) of rock, and can be used as indexes for predicting rock instability.

## Conclusion

To study the influence of cyclic stress on nonlinear behavior in saturated sandstone, the residual strain properties and energy dissipation characteristics of sandstone under tiered cyclic loading were experimentally investigated. The axial and radial deformations and energy dissipations evolution characteristics of sandstone under cyclic loading were analyzed. In combination with the mathematical statistics, the fluctuation coefficients for the residual strain and energy dissipation, and correlation coefficients for the axial/radial residual strains and energy dissipations were defined to describe this process. The results showed that: with the increase of cycle number in each cycle stage, the values of Δ*ε*_*p*_ and Δ*U*_*d*_ initially decreased rapidly, and then fluctuated near the cyclic stability line before the destruction stage. With the increase of the cycle stage, the fluctuation coefficients of Δ*ε*_*p*_ and Δ*U*_*d*_ decreased first, and then increased. The correlation coefficients of the axial-radial Δ*ε*_*p*_ and Δ*U*_*d*_ decreased first, and then increased. As mentioned above, the newly defined physical variables (*ξ*, *χ*, *ρ*) were closely related to the elastic-plastic state (or instability failure state) of the rock. Thus, they can be used as indexes for predicting rock instability.

## Supporting information

S1 Data (ZIP)
